# Alteration of Metabolites Accumulation in Maize Inbreds Leaf Tissue under Long-Term Water Deficit

**DOI:** 10.3390/biology10080694

**Published:** 2021-07-21

**Authors:** Natalija Kravic, Vojka Babic, Jelena Vukadinovic, Danijela Ristic, Vesna Dragicevic, Snezana Mladenovic Drinic, Violeta Andjelkovic

**Affiliations:** Maize Research Institute Zemun Polje, Slobodana Bajica 1, 11185 Belgrade-Zemun, Serbia; vbabic@mrizp.rs (V.B.); mesarovicj16@gmail.com (J.V.); dristic@mrizp.rs (D.R.); vdragicevic@mrizp.rs (V.D.); drinicsnezana2@gmail.com (S.M.D.); violeta@mrizp.rs (V.A.)

**Keywords:** anthocyanins, chlorophyll, drought, flavonols, grain yield, phenolic acids, *Zea mays* L.

## Abstract

**Simple Summary:**

As sessile organisms, plants are constantly exposed to diverse environmental stresses of which water deficit is the most significant because it limits plant growth, development, and productivity. In this work, we showed the influence of non-irrigation treatment on changes in maize leaf metabolite content. We argued that the different susceptibility of maize inbred lines to long-term water deficit will result in different patterns of change in metabolite accumulation. We emphasized the need for the careful interpretation of the level and type of accumulated metabolites in order to assess the drought tolerance status of maize inbred lines in terms of improved grain yield exhibited under severe water deficit conditions. Leaf metabolites that have contributed to higher grain yield under the condition of long-term water deficit could be considered as biochemical markers useful in breeding drought-tolerant maize.

**Abstract:**

Plants reconfigure their metabolic pathways to cope with water deficit. The aim of this study was to determine the status of the physiological parameters and the content of phenolic acids in the upper most ear leaf of maize inbred lines contrasting in drought tolerance in terms of improved plant productivity e.g., increased grain yield. The experiment was conducted under irrigation and rain-fed conditions. In drought-tolerant lines, the effect of water deficit was reflected through a chlorophyll and nitrogen balance index increase followed by a flavonols index decrease. The opposite trend was noticed in drought susceptible inbreds, with the exception of the anthocyanins index. Moreover, in comparison to irrigation treatment, opposite trends in the correlations between grain yield and physiological parameters found under water deficit conditions indicated the activation of different metabolic pathways in defense against water deficit stress. Concerning phenolic acid content, water deficit caused the reduction of protocatechuic, caffeic, and sinapic acid in all inbreds evaluated. However, the highly pronounced increase of ferulic and especially cinnamic acid content under water deficit conditions indicated possible crucial role of these secondary metabolites in preventing the harmful effects of water deficit stress, which, in turn, might be useful in maize breeding selection for drought tolerance.

## 1. Introduction

In temperate regions worldwide, ongoing climatic changes have caused frequent and severe summer droughts that seriously impair maize grain yield. The activation of specific physiological and molecular responses as plant mechanisms for acclimation and adaptation to challenging environmental conditions leads to changes in plant metabolism in order to minimize stress-induced damage. Plants grown under defined abiotic stress conditions such as water deficit, salinity, high and low temperatures, light, ozone, metal, or other individually applied stresses alter gene expression in a very specific and different way [1]. These alterations in gene expression result in a specific regulation of the metabolome, depending upon the particular stress, the plant species and its developmental stage [2].

The plant metabolome comprises a huge diversity of metabolites, and it is usually divided into the primary and secondary metabolism [3]. Phenylpropanoids and their derivatives such as phenolic acids, flavonoids, and anthocyanins are an important group of secondary metabolites that are essential for plant acclimation and survival to varying environmental conditions [4]. A variety of physiological roles have been attributed to these secondary metabolites, including scavenging of reactive oxygen species (ROS), enzyme activation, photoprotection, and signal regulation [5].

Besides the triggering acclimation and adaptation mechanisms, water deficit may also result in various damages [6]. Most damage-related parameters that can be measured in the field are indicators of leaf chlorophyll content, analysis of which provides a rapid and accurate technique for detecting and quantifying plant tolerance to drought stress [7,8].

In field-grown maize, detected identical genomic regions mapped as both agronomic and metabolic quantitative trait loci indicated the utility of metabolic traits for breeding selection [9,10,11].

The effect of water deficit on maize yield is particularly harmful during the reproductive stage, i.e., between flowering and early grain filling [12]. Both, assimilates supply to the kernel (e.g., source) and the sink potential of the kernel, determines the maize grain yield. Previously, it has been reported that drought had the most dramatic effect on the metabolite composition and source capacity of leaves compared to other organs [13,14,15]. However, maize breeding under temperate conditions has paid more attention to the sink potential, particularly under stress conditions [16]. Since the source ability is closely related to leaf metabolism, particularly under stress conditions, and due to the fact that grain is more susceptible to water stress than vegetative tissues, the prediction of grain yield from the physiological parameters of the leaves is a challenge. Accordingly, the aim of this study was (i) to determine whether the tolerance/susceptibility to water stress can be attributed to the level of the chlorophyll, flavonols, and anthocyanins as well as the phenolic acids in maize leaves and (ii) to evaluate the relationship between these metabolites and grain yield as an indicator of plant productivity by comparing plant performance under irrigation and rain-fed conditions.

## 2. Materials and Methods

### 2.1. Plant Material

In a two-year field trial, the entire collection (of ~6000 maize accessions) of the Maize Research Institute Zemun Polje (MRIZP) gene bank was screened for drought tolerance under controlled drought (e.g., managed stress environment—MSE) in Egypt. Secondary traits considered as indices of drought tolerance (i.e., the anthesis-silking interval—ASI, leaf rolling, staying green, barrenness, seed set, and grain filling) were monitored. An additional two-year evaluation for grain yield and general combining ability was conducted in the temperate conditions of Serbia and Macedonia. As a result, a drought-tolerant mini-core collection of 41 accessions (15 inbred lines, 13 local and 13 introduced maize landraces) was established.

In this study, three inbreds from the MRIZP gene bank drought tolerant mini-core collection (DT1, DT2, and DT3) and two drought sensitive inbred lines (DS1 and DS2) were evaluated.

### 2.2. Field Trial

The open-field experiment was conducted in 2017 at Zemun Polje, Serbia (44°52′00″ latitude N, 20°19′00″ longitude E, 81 m altitude) on Calcaric Chernozem of silty loam texture and set up according to randomized complete block design (RCBD). Sowing took place on April the 12th. Each genotype was sown in two rows with twenty plants per row. Intra-row and inter-row separation was 0.20 m and 0.75 m, respectively. Inbred lines were grown in two sets of field experiments: under irrigation treatment—I_75_ (i.e., 75% of full irrigation which was considered as the optimal condition—OC) and under non-irrigation treatment—I_0_ (which was considered as water deficit condition—WDC), with two replications per experimental set. In the I_75_ treatment, irrigation was applied when the soil water content reached approximately 60% of the total available water to the effective root zone depth (to 0.60 m) and took place on June the 21st (40 L m^−2^), July the 12th (50 L m^−2^), and August the 19th (40 L m^−2^).

Plants were manually harvested on September the 21st, and after drying to 14% water content, the yield was determined and presented as the average grain yield per plant (g plant^−1^).

### 2.3. Growth Conditions

During the experimental period in 2017, the average temperature during the vegetative period (April–September) was optimal (21 °C), while the flowering stage for the genotypes evaluated herein (i.e., last ten days in June and first ten days in July) was characterized by average temperatures of 27.8 °C and 30.3 °C, for the last ten days of June and the first ten days of July, respectively. The excessive amount of precipitation during the entirety of May (85.1 L m^−2^) contributed to shallow rooting and was followed by an extremely low amount of precipitation during the two subsequent crucial phenophases—the flowering phase (twenty-day period in June–July with only 17.4 L m^−2^ of rainfall) and the grain filling phase (the entirety of the July–August period with 45.9 L m^−2^ of rainfall). Since the general estimation of precipitation for optimal maize growth in the region where the trial was performed amounts to 459.0 L m^−2^ [17], the water deficit stress during the experiment could be considered as long-term and severe, particularly because of the previously developed shallow root system. The climatic parameters during the vegetative period for 2017 experiment and 20-yr average are given in Table 1.

### 2.4. Physiological Parameters (Nitrogen Balance Index, Chlorophyll, Flavonols and Anthocyanins Indices)

At flowering, the nitrogen balance index (NBI), chlorophyll index (Chl), flavonols index (Flav) and anthocyanins index (Anth) were analyzed using the Dualex Scientific (Force-A, Orsay, France) hand-held optical leaf clip sensor for non-destructive relative chlorophyll quantification. The measurements were conducted on the basal, middle, and apical position of the uppermost ear leaf and the obtained values were averaged. For each experimental set, leaf samples were taken from twenty randomly chosen plants per genotype in two replications. The same leaf samples were used for determination of phenolic acid content. 

### 2.5. Samples Preparation and Extraction of Free Phenolic Acids (PA)

For the extraction of the phenolic acids, 2 g of fresh leaf tissue was extracted twice with 3 mL MeOH/H_2_O (8:2, *v*/*v*) for 45 min in an ultrasonic bath [18]. The extracts were centrifuged at 3000 rpm for 5 min and filtered using a 0.45-μm PTFE membrane filter and directly injected into the HPLC. All of the chemicals used were of HPLC grade.

#### Quantification of PA

Separation of six phenolic acids (sinapic, cinnamic, protocatechuic, caffeic, *p*-coumaric, and ferulic) was performed on the analytical column Acclaim Polar Advantage II^®^ C18 (150 mm × 4.6 mm, 3 μm) obtained from Thermo Fisher Scientific at 25 °C. The mobile phase flow rate was 0.8 mL min^−1^ and contained 0.1% aqueous formic acid solution (A) and pure methanol (B). The linear gradient program was run from 0–10 min, 15–45% B; 10–25 min, 45–65% B; 25–30 min, 65–100% B; and 30–35 min, 100–15% B. The injection volume of each sample was 5 μL. The UV detection wavelengths were set at 278, 280, 290, and 300 nm. The PA content was presented as the mean value of the three independent injections, and it was expressed as μg g^−1^ fresh weight (FW). The Chromeleon software package (version 7.2) was used for instrument control as well as for data acquisition and analysis.

### 2.6. Statistical Analyses

The results were statistically analyzed using s two-way factorial RCBD analysis of variance (ANOVA) and presented as mean ± standard deviation (SD). For each trait, the coefficient of variation (CV) was determined. Significant differences between the genotype means were determined using the Fisher’s least significant difference (LSD) test at the 0.05 probability level, and differences with *p* ≤ 0.05 were considered as significant. SPSS software for Windows, version 14.0 (SPSS Inc., Chicago, IL, USA), was used for the statistical analyses. Pearson’s correlation coefficient was used to determine the relationship between the measured metabolites and the obtained grain yield.

## 3. Results

### 3.1. Physiological Parameters (NBI, Chlorophyll, Flavonols and Anthocyanins Indices)

According to ANOVA, all sources of variation (i.e., inbred lines, irrigation treatments, and inbred line by irrigation treatment interactions) exhibited highly significant influence on the majority of the observed physiological parameters. However, the only significant effect (*p* ≤ 0.05) for anthocyanins index (Anth) was achieved in the inbred line by irrigation treatment interaction (Table 2 and Table S1).

**Figure 1:**
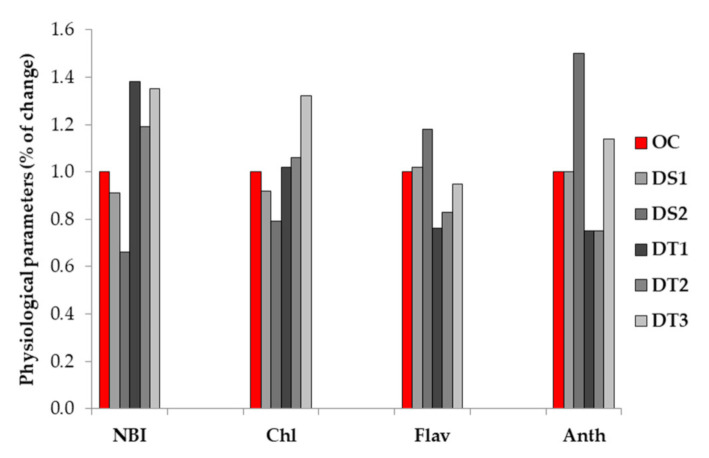
The percentage of changes for indices measured under water deficit stress (non-irrigation treatment—I_0_) compared to the optimal conditions (irrigation treatment—I_75_) evidenced in drought tolerant (DT1, DT2, and DT3) and drought susceptible (DS1 and DS2) inbreds. OC—values measured under optimal conditions given as 1 (100%).

Compared to the I_75_ irrigation treatment, water deficit (I_0_—non-irrigation treatment) caused the reduction of NBI in the drought susceptible (DS) inbreds DS1 and DS2 by 9% and 34%, respectively. However, under water deficit stress, drought tolerant (DT) inbreds exhibited a NBI increase, ranging from 19% to 35% to 38% for DT2, DT3, and DT1, respectively (Figure 1).

As with the NBI, the same trend of change in response to water deficit was observed for relative chlorophyll content. Under non-irrigation (I_0_) treatment, DS inbreds exhibited a Chl decrease of 8% and 22% in DS1 and DS2, respectively. Opposite to the DS inbred lines, the highest Chl increase of 32% was found in DT3, followed by slight Chl increase in the DT1 and DT2 lines (a 2% and 6% Chl increase, respectively) (Figure 1).

In comparison to the NBI and Chl performances, an opposite trend of change regarding relative flavonol content was observed in response to water deficit (Figure 1). Namely, the DS1 and DS2 lines exhibited a Flav increase of 2% and 18%, respectively. Among the drought tolerant inbreds, DT1 expressed the most pronounced Flav decrease of 24%, followed by DT2 and DT3 (a 17% and 5% Flav reduction, respectively).

Compared to the I_75_ treatment, the effect of the I_0_ treatment resulted in decreased Anth in the DT1 and DT2 lines (25% Anth reduction) or unchanged Anth in the DS1 inbred. However, a highly pronounced Anth increase of 50% was observed in the DS2 inbred, followed by smaller Anth increase of 14% exhibited by the DT3 inbred (Figure 1).

### 3.2. Free Phenolic Acids (PA) Content

According to ANOVA, both the effects of inbred line and irrigation treatment as well as the effects of their interaction were highly pronounced (*p* ≤ 0.001 and *p* ≤ 0.01) for the majority of the evaluated phenolic acids, with the exception of *p*-coumaric acid in response to irrigation treatment (*p* ≤ 0.05) (Table 3 and Table S2).

**Figure 2:**
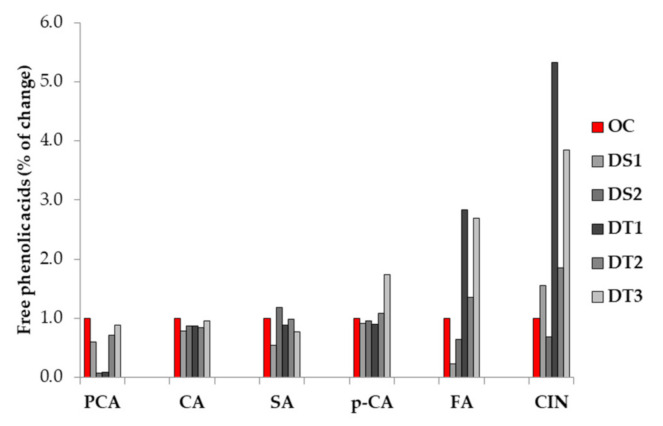
The percentage of changes for phenolic acids under water deficit stress (non-irrigation treatment—I_0_) compared to the optimal conditions (irrigation treatment—I_75_) evidenced in drought tolerant (DT1, DT2, and DT3) and drought susceptible (DS1 and DS2) inbreds. OC—values measured under optimal conditions given as 1 (100%).

Under optimal conditions, the DS lines exhibited lower protocatechuic acid (PCA) content in comparison to the DT lines. In response to water deficit (I_0_ treatment), all maize inbred lines exhibited decreased PCA content. In drought susceptible lines, PCA content declined by 40% (DS1) and 20% (DS2). A less pronounced PCA decrease was found in the DT inbreds, i.e., 10% in DT1, 28% in DT2, and 12% in DT3, respectively (Figure 2).

Under optimal conditions, the DS lines expressed significantly lower CA content in comparison to the DT lines. As with the PCA, the same trend of change in response to water deficit was found for caffeic acid (CA) content. Under I_0_ treatment, the decrease in CA content was more pronounced in the DS1 and DS2 lines (a 21% and 13% CA decrease, respectively). In the DT lines, CA content declined by 4%, 13%, and 16% in DT3, DT1, and DT2, respectively.

Concerning the changes in sinapic acid (SA) and cinnamic acid (CIN) content, the inbreds exhibited quite an opposite trend in response to water deficit. The majority of the inbreds showed a decline in SA content except for DS2 (an 18% SA content increase), the same genotype that experience a 30% decrease in CIN. While a decrease in SA content in response to water deficit (I_0_) treatment was as follows: DS2 > DT3 > DT1 (i.e., a 45%, 23%, and 12% decrease, respectively), the increase of CIN was determined as: DT1 > DT3 > DT2 > DS1 (i.e., a 432%, 284%, 85% and 56% increase, respectively).

In response to water deficit, a slight decrease in *p*-coumaric acid (*p*-CA) was found in both DS maize inbreds (an 8% and 4% increase) as well as in DT1 (a 10% increase). The other two DT inbreds displayed *p*-CA increases of 8% (DT2) and 74% (DT3).

Water deficit caused a decrease of ferulic acid (FA) content in the DS inbreds (77% and 35%) and an increase of FA content in the DT maize lines (184%, 34%, and 170%).

### 3.3. Grain Yield (GY)

**Figure 3:**
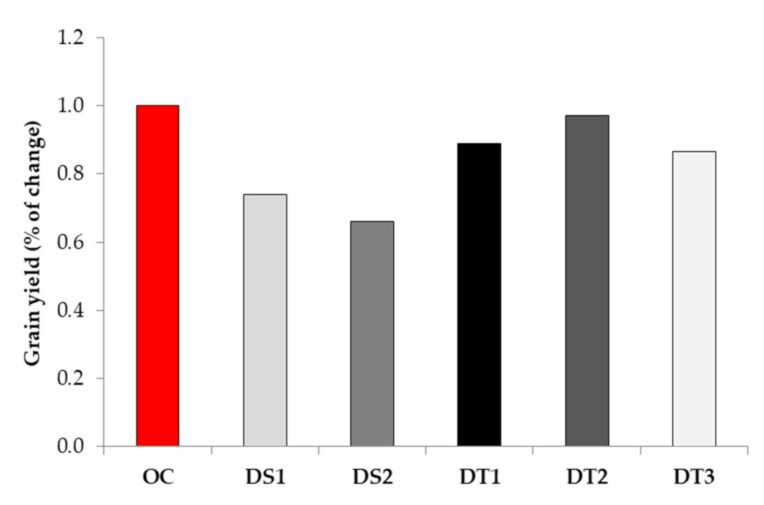
The percentage of changes for grain yield under water deficit stress (non-irrigation treatment—I_0_) compared to the optimal conditions (irrigation treatment—I_75_) evidenced in drought tolerant (DT1, DT2, and DT3) and drought susceptible (DS1 and DS2) inbreds. OC—values measured under optimal conditions given as 1 (100%).

Under I_75_ irrigation treatment, grain yield obtained from DS lines was 19.9 g plant^−1^ and 18.0 g plant^−1^ for DS1 and DS2, respectively. On the other hand, the DT lines achieved significantly higher grain yield, ranging from 34.1 g plant^−1^ (DT1) to 49.4 g plant^−1^ (DT3) to 54.0 g plant^−1^ (DT2). The percentage of change in grain yield in response to water deficit is displayed in Figure 3. In all of the evaluated genotypes, water deficit resulted in a grain yield decrease, ranging from 26% in drought susceptible DS2 to 47% in DS1; in drought tolerant inbreds, grain yield decline was as follows: 11%, 3%, and 14% for DT1, DT2, and DT3, respectively.

### 3.4. Correlations

For both the I_75_ and I_0_ field experimental sets separately, Pearson’s correlations were used to determine the relationship both within and between the evaluated metabolites.

The same trend in the correlations within the majority of the physiological parameters was observed for both experimental sets and was more pronounced under the I_0_ treatment. The exception was the opposite trend in the correlation between Chl and Flav (Table 4).

Additionally, the same trend in the correlations within the majority of the phenolic acids evaluated was found in both experimental sets. However, FA was the only phenolic acid responsible for the opposite trend in the correlations, which was evidenced in relation to PCA, CA, SA, and *p*-CA (Table 3).

In response to applied irrigation treatment, evaluation of the relationship between the physiological parameters (i.e., indices) and the phenolic acids revealed highly pronounced opposite trend in correlations. However, the same trend in the correlations for both experimental sets was mostly related to correlations with FA (Table 4).

**Figure 4:**
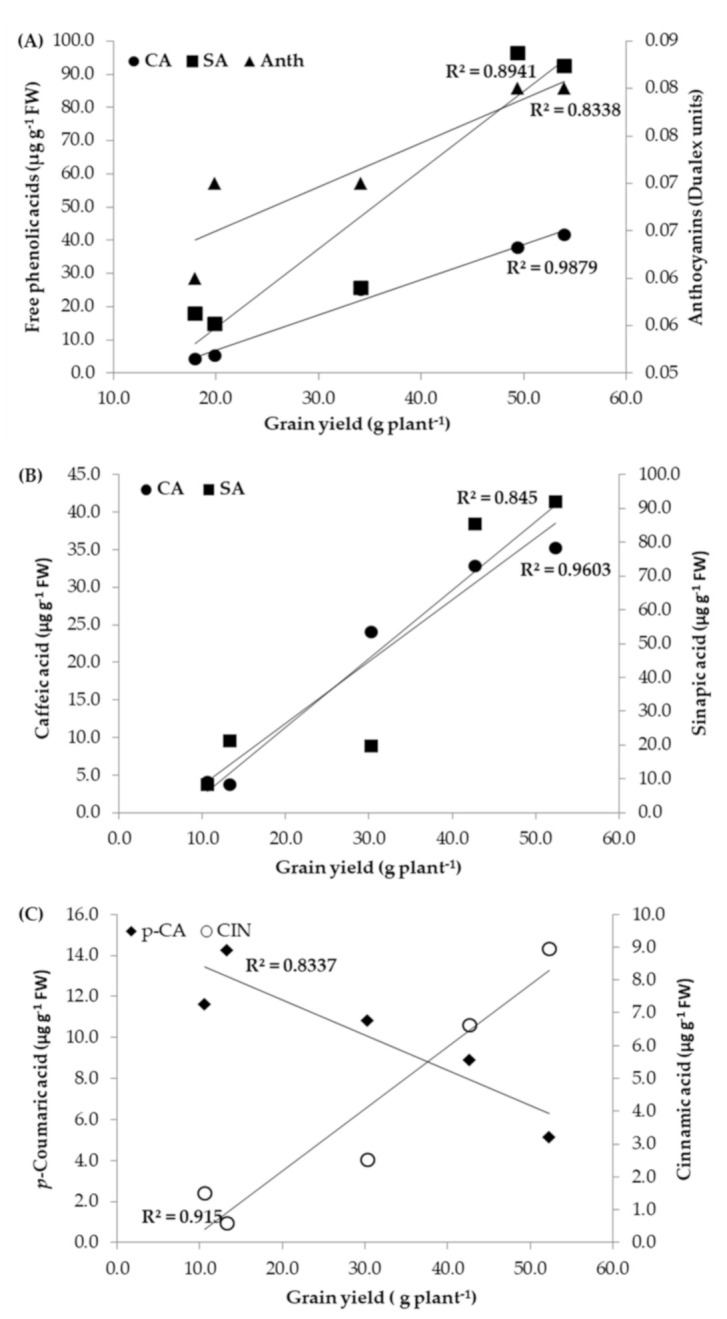
Pearson regression coefficients for the correlations: (**A**) between grain yield and the anthocyanins index and caffeic (CA) and sinapic acid (SA) obtained under I_75_ irrigation treatment; (**B**) between grain yield and caffeic (CA) and sinapic acid obtained under I_0_–non-irrigation treatment; (**C**) between grain yield and *p*-coumaric acid (*p*-CA) and cinnamic acid (CIN) obtained under I_0_–non-irrigation treatment.

For both I_75_ and I_0_ field experimental sets separately, Pearson correlations were also used to determine the relationship between the grain yield and the physiological parameters, i.e., phenolic acids. Under optimal (I_75_) conditions, correlations between GY and NBI, and GY and Chl were negative (r = −0.534 and r =−0.245; non-significant correlations), while they were positive between GY and Flav (r = 0.701; non-significant correlation) and GY and Anth (r = 0.913, *p* ≤ 0.05) (Figure 4A). Under water deficit (I_0_) conditions, the opposite trend in the correlations between GY and all other physiological parameters was as follows: NBI: r = 0.863; Chl: r = 0.765; Flav: r = −0.788, Anth: r = −0.720; non-significant correlations.

Under both I_75_ and I_0_ treatments, trend in correlations between GY and the majority of PA were the same, except between GY and FA (under I_75_: r = −0.301; under I_0_: r = 0.869; non-significant correlations). The most significant positive correlations obtained under optimal conditions were between GY and CA (r = 0.994, *p* ≤ 0.001) and between GY and SA (r = 0.946, *p* ≤ 0.05), displayed in Figure 4A. Under water deficit conditions, GY achieved the most significant correlations with CA (r = 0.980, *p* ≤ 0.01) and SA (r = 0.919, *p* ≤ 0.05) (Figure 4B) as well as with CIN (r = 0.957, *p* ≤ 0.01) and *p*-CA (r = −0.919, *p* ≤ 0.05), presented in Figure 4C.

## 4. Discussion

### 4.1. Physiological Parameters (NBI, Chlorophyll, Flavonols and Anthocyanins Indices)

Water deficit has been reported to diminish root nutrient uptake and translocation to the leaves [19]. As a highly energy demanding process [20], nitrogen (N) assimilation, especially the reduction of nitrate in leaves under water deficit, could use the excessive reducing power derived from photosynthesis [21]. In such a way, N assimilation acts as an important alternative sink of electron and excessive excited energy in order to minimize the photoinhibition and photodamage of photosynthesis and to stimulate CO_2_ assimilation under the conditions of stomatal limitation imposed by drought [22]. Since non-destructively measured leaf Chl content was reported to be an indicator of N nutrition of crops [23] while NBI is more of an indicator of C/N allocation changes due to N-deficiency than a measure of leaf nitrogen content [24], the results in our study, i.e., the reduced NBI in DS lines and increased NBI in DT lines in response to water deficit, suggest that there is a sufficient N supply in the DT lines to facilitate the assimilation of stored nitrates under water deficit conditions, which could partly contribute to mitigating the photoinhibition of photosynthesis caused by water deficit stress.

Moreover, the Chl decrease exhibited by DS inbreds and the Chl increase observed in DT inbreds in response to water deficit revealed that the components of the photosynthetic apparatus could be damaged in DS lines and that the DT lines could possess the adaptability to decrease/evade impairment resulting from water stress. It can be suggested that genetic differences exist in the reaction of the photosynthetic apparatus to drought and that in drought tolerant genotypes, the photosynthetic process has a higher tolerance to water deficit stress [25].

It has been reported that flavonoids, a class of specialized secondary metabolites, including flavonols and anthocyanins with strong radical scavenging activity, contribute to the mitigation of oxidative and drought stress in plants [26]. In our experiment, in response to water deficit, opposite trend regarding relative flavonol content was observed between the DS lines (i.e., Flav increase) and the DT lines (i.e., Flav decrease). Our results are in agreement with the findings that plants under stress conditions often produce a higher degree of flavonoids compared to non-stressed plants [27]. In addition, studies on a series of *Arabidopsis* lines both over accumulating and lacking flavonoids under MYB overexpression, which are required to identify flavonoid function, showed that flavonoid accumulation tends to decrease water loss [28]. Accordingly, increased Flav in the DS lines indicated the existence of s compromise between photosynthesis and transpiration. By closing the stoma to conserve water, the diffusion of atmospheric CO_2_ into the leaf for carbon fixing is compromised. Therefore, productivity in the DS inbreds is limited by the need to retain water.

Under water stress, nitrogen deficit impairs photosynthetic function and efficiency and decreases the levels of Calvin cycle enzymes, which results in the induced or enhanced accumulation of foliar anthocyanin in leaves of many plant species [29]. Highly pronounced increases in Anth found in DS2, the same line with the highest reduction of both Chl and NBI, suggests that N deficiency caused by water deficit triggered the expression of gene encoding enzymes associated with anthocyanin biosynthesis [30].

### 4.2. Free Phenolic Acids (PA) Content

It has been reported that drought stress regulates the phenylpropanoid biosynthetic pathways of phenolic acids, leading to an enhanced accumulation of these compounds, which act as antioxidants and prevent plants from the adverse effects of water deficit conditions [31]. A highly increased accumulation of ferulic acid in DT inbred lines evaluated herein (from 36% in DT1 to 170% in DT2 to 184% in DT3) may indicate the activation of defense reactions in these lines. The increased ferulic acid may originate from the activation of enzyme phenylalanine ammonia-lyase (PAL), the key enzyme in the biosynthesis of simple phenolic acids in plants, strategically located at a branching point between the primary and secondary metabolism [32]. Our findings are consistent with reported the increased PAL activity and the accumulation of high levels of ferulic acid in some winter triticale drought-tolerant genotypes [33]. The observed ferulic acid increase, as an effect of a decrease of the water potential in leaves, could be the result of protective mechanisms being triggered in the DT maize inbred lines and could be an indicator of the resistance to drought stress [34].

In response to water deficit, the enhanced biosynthesis of cinnamic acid in leaves was reported for tolerance in peanut [35,36] and tobacco [37]. These findings are in line with ours, which show that long-term drought resulted in a highly pronounced cinnamic acid content increase in the DT maize inbreds (from 85% in DT2 to 284% in DT3 to 432% in DT1). It was reported that cinnamic acid helps to reduce lipid peroxidation and regulate the activities of ROS-scavenging enzymes, i.e., increases the activities of antioxidant enzymes such as superoxide dismutase (SOD) and peroxidase (POD) in *Zea mays* L. [38] and cucumber leaves [39].

On the other hand, some reports indicated that water deficit declined the phenolic content of plant tissues. In studies on *Rehmannia glutinosa* seedlings, it has been shown that in response to water deficiency treatment, the content of protocatechuic acid, caffeic acid, sinapic acid, *p*-coumaric acid, and ferulic acid significantly decreased, suggesting that the decrease in the phenolic acids might result from a decline in the activity of key enzymes related to their biosynthesis [40]. In our experiment, long-term and continuous water stress inhibited the biosynthesis of protocatechuic acid, caffeic acid, and sinapic acid in maize inbreds leaves and was much more pronounced in the DS inbreds; however, accumulation of *p*-coumaric acid and ferulic acid was only impaired in the DS lines. Our results are, to a higher extent, in line with the results obtained under a long-term drought study on *Vitis vinifera* leaves [41]. The decreased content of some of the phenolic acids in the DT maize inbred lines could be considered as a slowdown in these secondary metabolites during long-term drought stress. As such, the plant reduces energy expenditure until the end of the activity stressor. This could be observed as a survival strategy in disadvantaged environment conditions.

### 4.3. Grain Yield (GY)

The responses of plants to water deficit observed under field conditions are generally much more complex than those measured under controlled laboratory conditions because of the other factors accompanying the water deficit that influence the nature of the stress response [42]. The impact of water deficit on maize grain yield depends on the synchrony between the stress and the developmental stage. If it occurs during flowering, as was the case in this study, yield losses are highly pronounced [43]. Differences in performance and yield potential could be associated with the variability in quantitative traits and processes, which are more expressed under stress conditions. It has previously been reported that yield potential (including heterosis) is a constitutive trait and that the average grain yield reduction in maize hybrids under drought compared to well-watered conditions is up to 20% [44]. Compared to optimal I_75_ irrigation treatment, the response of individual DT lines to water deficit was far below this threshold, e.g., decrease in grain yield ranged from 3% in DT2 to 11% and 14% in the DT1 and DT3 inbreds, respectively, while the DS lines exhibited a reduced grain yield of 26–47%. A high yield potential could be achieved under optimal conditions and conditions of mild environmental stress; however, under more intensive stress (the long-term water deficit stress in the present study), only the germplasm with stress-adaptive genes maintained a stable yield [45].

### 4.4. Correlations

In all correlations within physiological parameters, the indices were more pronounced under long-term water deficit in the field. Positive correlations between NBI and Chl confirmed that the nitrate uptake, allocation, and assimilation in plants were closely associated with plant tolerance to adverse water deficit conditions [46] and that the majority of the assimilated N in plants is invested in photosynthetic machinery [47,48]. On the other hand, negative correlations between NBI and Flav confirmed that the nitrate uptake, allocation, and assimilation in plants may regulate the biosynthesis of flavonoids by controlling the C flow allocation between the primary and secondary metabolism [49]. However, a negative correlation between Chl and Flav only observed under I_0_ treatment implies the reported tendency of flavonoid accumulation to decrease water loss from the transpirational pathways [28], which was exhibited by the DS inbreds, and decrease changes in the photosynthetic apparatus in response to water deficit in terms of decreased CO_2_ assimilation and net photosynthesis due to a reduced stomatal opening [50].

Caffeic acid mediates the absorption of high energy radiation in mesophyll cells under drought stress, and this mechanism involves the production of ferulic acid through the methylation of caffeic acid; thus, enhancing the drought resistance [51]. It can be concluded that under a long-term field water deficit during flowering, by exhibiting significantly higher level of free ferulic acid in comparison to the DS lines, the DT maize inbreds protect photosynthetic machinery, which would be otherwise disrupted by the high energy radicals produced due to disturbed water relations under drought stress, which is in agreement with studies on triticale genotypes differing in drought tolerance [33]. As reported for the above-ground biomass of wheat, positive correlations between caffeic acid and ferulic acid and cinnamic acid as well as between cinnamic acid and sinapic acid obtained under I_0_ treatment confirmed the involvement of caffeic acid in drought management, which was additionally strengthened by the synthesis of other phenolic acids under drought stress, such as cinnamic acid, sinapic acid, and *p*-coumaric acid, which may contribute to the protection of the photosynthetic organs against oxidative stress and dehydration of the leaves [52].

Although the flowering period in maize is the phenophase that is the most vulnerable to water deficit, water and nitrogen availability during the grain-filling period determine the extent to which sink and source contribute to yield formation, and limited resource availability will mainly result in source restrictions by reducing current photosynthesis, which is not as likely with sink limitations [53]. Since chloroplasts comprise more than 70.0% of the nitrogen taken up from the environment, grain yield and its quality depend upon chloroplast breakdown as a consequence of water stress and the remobilization of nitrogen. Improved grain yield obtained by the DT lines under long-term field drought imply their better preserved light-harvesting Chl–protein complex in the thylacoid membranes of the chloroplasts, which are rapidly catabolized under stress conditions, strongly suggesting that it may be a readily mobilized source of amino nitrogen for the maintenance of protein synthesis as well as a source of carbon skeletons for energy production during stress [54]. This is confirmed with positive correlations between GY and NBI and GY and Chl obtained under severe long-term water deficit stress. According to Tremblay et al. [23] and Cartelat et al. [24], positive correlations between NBI and Chl and the majority of phenolic acids as well as between GY and caffeic acid, sinapic acid, and cinnamic acid obtained under I_0_ treatments additionally confirmed the protective role of phenolic acids in mitigating the photoinhibition of photosynthesis caused by water deficit stress and in stimulating CO_2_ assimilation under conditions of stomatal limitation imposed by drought.

## 5. Conclusions

The development of drought-tolerant genotypes is of utmost importance for plant breeding. Further progress in maize performance under drought is expected by combining marker-assisted breeding with metabolite markers. Hence, the metabolites from maize leaves subjected to long-term water deficit in field were analyzed in this study. Studies on metabolites using field samples are still rare, and to our knowledge, this is one of the very few studies reporting metabolite responses to stress in a field-grown crop, making our results a good reference for future studies. It was observed that the distribution of the Dualex measured values for the Chl index and especially for the NBI among the evaluated maize inbred lines is consistent with their drought tolerance/susceptibility classification. In addition, opposite trends in the correlations between grain yield and all of the physiological indices found under water deficit conditions in comparison to well-watered field conditions indicated the activation of different metabolic pathways in defense against existing severe water stress. The HPLC analysis of the field stress maize leaf samples successfully identified metabolites closely related to grain yield under water deficit stress conditions. Caffeic acid, sinapic acid, and especially cinnamic acid are quite promising metabolic markers for maize breeding, as those in the well-watered condition were correlated to grain yield in water deficit conditions, allowing selection under optimal growth conditions. Further trials should be conducted to confirm the relationship between these metabolic traits and yield performance under stress as well as to test the effectiveness of the metabolites for the biochemical maker-assisted breeding for drought-tolerant maize.

## Figures and Tables

**Table 1:** Difference in climate variables between vegetative period for 20-yr average (1997–2016) and for the 2017 experiment.

Month	Temperature [°C]		Precipitation [L m^−2^]	
	20-yr Avg.	2017	20-yr Avg.	2017
April	13.5	−0.8	46.1	5.7
May	18.3	0.1	63.4	21.7
June	21.7	2.6	86.0	−33.0
July	23.6	2.3	77.6	−51.2
August	23.3	2.8	62.6	−43.1
September	18.1	0.3	66.1	−20.3
April–Sept.	19.8	1.2	67.0	−20.0

**Table 2:** Analysis of variance for physiological parameters (i.e., indices) evaluated in maize inbred lines under different water regimes with two replications per treatment. Abbreviations: NBI: nitrogen balance index; Chl: chlorophyll index; Flav: flavonols index; Anth: anthocyanins index; SV: source of variation; IL: inbred line; T: irrigation treatment; d.f.: degree of freedom; MS: mean square; ns: non-significant.

Trait	SV	df	MS	*p*
NBI	IL	4	27.247	≤0.01
	T	1	39.931	≤0.001
	IL × T	4	145.139	≤0.001
Chl	IL	4	18.564	≤0.001
	T	1	0.169	≤0.001
	IL × T	4	71.678	≤0.001
Flav	IL	4	0.020	≤0.001
	T	1	0.023	≤0.001
	IL × T	4	0.042	≤0.001
Anth	IL	4	0.000	ns
	T	1	0.000	ns
	IL × T	4	0.000	≤0.05

**Table 3:** Analysis of variance for phenolic acids evaluated in maize inbred lines under different water regimes with two replications per treatment. Abbreviations: PCA: protocatechuic acid; CA: caffeic acid; SA: sinapic acid; *p*-CA: *p*-coumaric acid; FA: ferulic acid; CIN: cinnamic acid; SV: source of variation; IL: inbred line; T: irrigation treatment; d.f.: degree of freedom; MS: mean square.

Trait	SV	df	MS	*p*
PCA	IL	4	1650.324	≤0.001
	T	1	425.872	≤0.001
	IL × T	4	30.262	≤0.01
CA	IL	4	1076.958	≤0.001
	T	1	40.442	≤0.001
	IL × T	4	0.988	≤0.01
SA	IL	4	6565.992	≤0.001
	T	1	89.465	≤0.01
	IL × T	4	31.525	≤0.01
*p*-CA	IL	4	52.949	≤0.001
	T	1	1.260	≤0.05
	IL × T	4	5.526	≤0.001
FA	IL	4	0.352	≤0.001
	T	1	0.255	≤0.001
	IL × T	4	0.875	≤0.001
CIN	IL	4	26.190	≤0.001
	T	1	27.028	≤0.001
	IL × T	4	5.636	≤0.001

**Table 4:** Pearson correlation coefficients for correlations within and between metabolites observed under I_75_ (above the diagonal) and I_0_ (below the diagonal) irrigation treatments. NBI: nitrogen balance index; Chl: chlorophyll index; Flav: flavonols index; Anth: anthocyanins index; PCA: protocatechuic acid; CA: caffeic acid; SA: sinapic acid; *p*-CA: *p*-coumaric acid; FA: ferulic acid; CIN: cinnamic acid; * and ** are significant at the *p* ≤ 0.05 and *p* ≤ 0.01 probability levels, respectively.

Trait	NBI	Chl	Flav	Anth	PCA	CA	SA	*p*-CA	FA	CIN
NBI	–	0.856	−0.269	−0.653	−0.803	−0.586	−0.340	0.666	0.662	0.032
Chl	0.877 *	–	0.235	−0.369	−0.828	−0.308	0.040	0.678	0.515	0.011
Flav	−0.910 *	−0.621	–	0.567	0.151	0.696	0.812	−0.205	−0.404	0.151
Anth	−0.715	−0.317	0.920 *	–	0.563	0.889 *	0.879 *	−0.698	−0.139	0.599
PCA	0.825	0.966 **	−0.595	−0.247	–	0.706	0.359	−0.943 *	−0.668	0.329
CA	0.942 *	0.849	−0.839	−0.712	0.773	–	0.912 *	−0.825	−0.399	0.637
SA	0.695	0.492	−0.695	−0.769	0.292	0.863	–	−0.550	−0.115	0.656
*p*-CA	−0.772	−0.572	0.856	0.855	−0.489	−0.868	−0.838	–	0.441	−0.624
FA	0.846	0.746	−0.684	−0.593	0.573	0.906 *	0.859	−0.637	–	0.333
CIN	0.773	0.561	−0.817	−0.852	0.415	0.906 *	0.957 *	−0.960 *	0.783	–

*n* = 5 (five inbred lines per irrigation treatment).

## Data Availability

The data presented in this study are available in the article.

## Supplementary Materials

The following are available online at https://www.mdpi.com/article/10.3390/biology10080694/s1, Table S1: The effect of two water regimes on the relative content of physiological parameters (i.e., indices) evaluated in drought susceptible (DS) and drought tolerant (DT) maize inbred lines. Table S2: The effect of two water regimes on phenolic acids content evaluated in drought susceptible (DS) and drought tolerant (DT) maize inbred lines.
