# Supplementary material for: Alteration of Metabolites Accumulation in Maize Inbreds Leaf Tissue under Long-Term Water Deficit
Source: Biology (Basel). 2021 Jul 21;10(8):694. doi: 10.3390/biology10080694 (PMC8389289; doi:10.3390/biology10080694)
Supplement: Supplementary file 1 [file biology-10-00694-s001.zip › Table S1.pdf]

Table S1. The effect of two water regimes on relative content of physiological parameters (*i.e.* indices) evaluated in drought susceptible (DS) and drought tolerant (DT) maize inbred lines. For each water regime, the results are mean ± SD of duplicate measurement, on 20 plants per replication. Means followed by the same letter within the same column are not significantly different ($p \leq 0.05$). Abbreviations: SV – source of variation; NBI – nitrogen balanse index; Chl – chlorophyll index; Flav – flavonols index; Anth – anthocyanin index; OC – optimal conditions (*i.e.* I_{75} – irrigation treatment); WDC – water deficit conditions (*i.e.* I_0 – non-irrigation treatment); LSD – Least Significant Difference; CV – coefficient of variation.

**SV**	**NBI**	**Chl**	**Flav**	**Anth**
DS1	36.89 ± 1.75c	41.19 ± 1.79c	1.110 ± 0.04b	0.070 ± 0.01a
DS2	37.40 ± 2.35bc	46.15 ± 1.74a	1.263 ± 0.04a	0.073 ± 0.01a
DT1	42.28 ± 2.92a	46.30 ± 2.98a	1.117 ± 0.03b	0.067 ± 0.01a
DT2	42.41 ± 2.92a	45.57 ± 1.91a	1.080 ± 0.07c	0.070 ± 0.01a
DT3	40.00 ± 1.55ab	43.78 ± 1.02b	1.120 ± 0.03b	0.075 ± 0.00a
$LSD_{0.05}$	2.621	1.260	0.016	0.016
DS1 × OC	38.57 ± 1.81c	43.00 ± 1.59e	1.100 ± 0.03ef	0.065 ± 0.01bc
DS1 × WDC	35.21 ± 1.69cd	39.38 ± 1.99fg	1.120 ± 0.04e	0.075 ± 0.01abc
DS2 × OC	45.02 ± 2.98b	51.68 ± 2.26a	1.155 ± 0.04d	0.055 ± 0.00c
DS2 × WDC	29.78 ± 1.72e	40.62 ± 1.21f	1.370 ± 0.04a	0.090 ± 0.01a
DT1 × OC	35.52 ± 1.63cd	45.82 ± 2.51cd	1.275 ± 0.02b	0.075 ± 0.01abc
DT1 × WDC	49.03 ± 4.20a	46.78 ± 3.44c	0.960 ± 0.05g	0.060 ± 0.01bc
DT2 × OC	38.70 ± 3.77c	44.25 ± 1.09de	1.180 ± 0.07c	0.080 ± 0.01ab
DT2 × WDC	46.13 ± 2.07ab	46.90 ± 2.73c	0.980 ± 0.08g	0.060 ± 0.00bc
DT3 × OC	34.10 ± 1.05d	37.78 ± 1.07g	1.150 ± 0.03d	0.070 ± 0.00abc
DT3 × WDC	45.90 ± 2.05ab	49.79 ± 0.96b	1.090 ± 0.04f	0.080 ± 0.01ab
$LSD_{0.05}$	3.706	1.781	0.023	0.023
CV(%)	4.12	1.77	1.54	14.24
