# Supplementary material for: Alteration of Metabolites Accumulation in Maize Inbreds Leaf Tissue under Long-Term Water Deficit
Source: Biology (Basel). 2021 Jul 21;10(8):694. doi: 10.3390/biology10080694 (PMC8389289; doi:10.3390/biology10080694)
Supplement: Supplementary file 1 [file biology-10-00694-s001.zip › Table S2.pdf]

Table S2. The effect of two water regimes on phenolic acids content (μg g^{-1} fresh weight) evaluated in drought susceptible (DS) and drought tolerant (DT) maize inbred lines. For each water regime, the results are mean ± SD of duplicate measurement, on 20 plants per replication. Abbreviations: SV – source of variation; PCA – protocatechuic acid; CA – caffeic acid; SA – sinapic acid; p-CA – p-coumaric acid; FA – ferulic acid; CIN – cinnamic acid; OC – optimal conditions (i.e. I_{75} – irrigation treatment); WDC – water deficit conditions (i.e. I_0 – non-irrigation treatment); LSD – Least Significant Difference; CV – coefficient of variation.

**SV**	**PCA**	**CA**	**SA**	***p*-CA**	**FA**	**CIN**
SD1	25.28 ± 2.58d	4.68 ± 0.55d	11.56 ± 1.29c	12.09 ± b	0.80 ± d	1.24 ± 0.15c
SD2	17.13 ± 1.27e	4.00 ± 0.39d	19.63 ± 1.34b	14.51 ± a	0.90 ± c	0.71 ± 0.11d
DT1	42.75 ± 3.22c	35.30 ± 2.55b	90.90 ± 6.17a	9.37 ± c	1.43 ± a	3.93 ± 0.23b
DT2	51.17 ± 3.97b	38.47 ± 2.16a	92.22 ± 4.90a	4.93 ± e	1.33 ± b	6.88 ± 0.77a
DT3	67.96 ± 4.34a	24.58 ± 1.92c	22.71 ± 2.24b	8.52 ± d	0.82 ± d	1.58 ± 0.13c
$LSD_{0.05}$	2.552	1.590	3.264	0.782	0.072	0.466
DS1 × OC	31.51 ± 3.52f	5.23 ± 0.53f	14.93 ± 1.53e	12.56 ± 1.27b	1.31 ± 0.13c	0.97 ± 0.16ef
DS1 × WDC	19.06 ± 1.64g	4.13 ± 0.57f	8.20 ± 1.05f	11.62 ± 0.99bc	0.30 ±0.11h	1.51 ± 0.15e
DS2 × OC	19.00 ± 1.37g	4.29 ± 0.44f	18.02 ± 1.34de	14.78 ± 1.28a	1.09 ± 0.16e	0.84 ± 0.12f
DS2 × WDC	15.26 ± 1.17h	3.71 ± 0.34f	21.24 ± 1.34cd	14.23 ± 1.56a	0.71 ± 0.13f	0.58 ± 0.11f
DT1 × OC	45.00 ± 2.67d	37.72 ± 2.83b	96.49 ± 6.79a	9.86 ± 0.75de	0.74 ± 0.13f	1.24 ± 0.10ef
DT1 × WDC	40.51 ± 3.77 e	32.88 ± 2.27d	85.32 ± 5.55b	8.88 ± 0.80e	2.11 ± 0.21a	6.62 ± 0.36
DT2 × OC	59.58 ± 4.35c	41.76 ± 2.76a	92.51 ± 5.24a	4.73 ± 0.34g	1.13 ± 0.14de	4.83 ± 0.76c
DT2 × WDC	42.76 ± 3.59de	35.17 ± 1.56c	91.93 ± 4.55a	5.12 ± 0.45fg	1.52 ± 0.10b	8.93 ± 0.79a
DT3 × OC	72.29 ± 4.69a	25.14 ± 2.21e	25.65 ± 2.45c	6.23 ± 0.46f	0.45 ± 0.07g	6.65 ± 0.11f
DT3 × WDC	63.64 ± 3.99b	24.02 ± 1.62e	19.76 ± 2.04d	10.81 ± 0.84cd	1.20 ± 0.08d	2.51 ± 0.14d
$LSD_{0.05}$	3.662	2.249	4.616	1.106	0.100	0.660
CV (%)	3.91	4.64	4.30	4.95	3.71	10.20
